# SIRT4 and SIRT6 Serve as Novel Prognostic Biomarkers With Competitive Functions in Serous Ovarian Cancer

**DOI:** 10.3389/fgene.2021.666630

**Published:** 2021-07-15

**Authors:** Huihuan Wang, Juan Li, Rui Huang, Lei Fang, Shan Yu

**Affiliations:** ^1^Department of Computer Science, School of Basic Medicine, Harbin Medical University, Harbin, China; ^2^Department of Pathology, Second Affiliated Hospital of Harbin Medical University, Harbin, China; ^3^Department of Colorectal Surgery, Second Affiliated Hospital of Harbin Medical University, Harbin, China; ^4^Department of Gynecology, Second Affiliated Hospital of Harbin Medical University, Harbin, China

**Keywords:** sirtuins family, ovarian cancer, prognostic biomarker, SIRT4, SIRT6

## Abstract

Sirtuins (SIRTs) are class III histone deacetylases (HDACs) that include seven members and are widely expressed in mammals. Accumulating evidence shows that sirtuins may have contradictory roles in various malignancies. They mainly participate in metabolic homeostasis, DNA damage repair, cell survival, and differentiation, as well as other cancer-related biological processes. To better understand their prognostic role and biological functions, we used comprehensive bioinformatic analyses to demonstrate the expression and mutation of sirtuin family member genes in ovarian cancer (OC), with a detailed focus on prognostic prediction, including the effectiveness of anti-OC drugs. Furthermore, the co-expression genes of SIRT4 and SIRT6 with contradictory survival prediction values in both overall and progression-free survival (PFS) times were further analyzed through Gene Ontology enrichment and Kyoto Encyclopedia annotation. Additionally, we performed and obtained the immunohistochemical staining patterns of these two biomarkers from the serous OC patient database and clinical patient samples to demonstrate their potential applicability in clinical pathology. According to our findings, SIRT4 and SIRT6 are novel prognostic biomarkers that may serve as contradictory competitors for OC cell survival. They are also sensitive biomarkers for the prediction of Avastin’s anticancer effect. While SIRT4 is related to the immune response during oocyte maturation, SIRT6 participates in immune-related disease pathways and mitochondrial metabolism-mediated DNA translation. These findings contribute to the novel hypothesis that SIRT4 and SIRT6 act as contradictory competitors in the regulation of OC behavior. Further studies are required to validate our hypothesis.

## Introduction

The modern era of cancer research has entered a new world of gene network analysis through bioinformatics-based tools centered on big data from multi-omic platforms. However, manipulating the expression of a single protein is still of great translational interest because of its unparalleled convenience for clinical translation. Despite advances in therapeutic technology, cancer remains the second most common cause of death worldwide. Metastasis and drug resistance are the key problems that result in patient deaths in the advanced stages of cancer. Ovarian cancer (OC) is the deadliest of all gynecological cancers, and it is the fifth leading cause of cancer-related deaths in women. Only 14.8% of OC patients are diagnosed at an early stage due to the absence of symptoms until later stages. Surgical treatment is believed to be the only way to cure this disease. However, most OC patients lose the opportunity to avail of surgical interventions as they are already at an advanced stage by the time they are diagnosed. Therefore, there is an urgent need to identify novel sensitive markers for the early detection of OC and the prediction of chemotherapy or targeted therapy effectiveness.

Sirtuins are homologs of the budding yeast silent information regulator two (SIRT) in mammals. There are seven sirtuins (SIRT1–SIRT7) constituting the sirtuin family of enzymes, also known as class III histone deacetylases (HDACs), and these are widely expressed in normal human tissues (Michishita et al., 2005). SIRT1–SIRT7 share a well-conserved nicotinamide adenine dinucleotide (NAD^+^)-binding catalytic domain. Sirtuins are divided into four classes based on their specificity and catalytic activity through the amino sequence. Notably, sirtuins have specific intracellular locations in human tissues for their physiological functions. SIRT1, SIRT6, and SIRT7 are primarily expressed in the nucleus or nucleolus (Ford, 2006; Mostoslavsky et al., 2006). SIRT2 is located in the cytoplasm, but can shuttle into the nucleus during mitosis (Vaquero, 2006). There are still many debates about the location of SIRT3, SIRT4, and SIRT5. According to most of the current findings, they are mainly expressed in the cytosol around the mitochondria (Huang et al., 2010). Evidence demonstrates that sirtuins play a crucial role in many diseases including cancers. They have been linked to human tumorigenesis *via* the regulation of metabolic homeostasis, DNA damage repair, and cell survival and differentiation. Dysregulated expressions of members of the sirtuin family are reported to be related to aerobic glycolysis, tumor angiogenesis, autophagy, and oxidative stress in human solid cancers (Rajendran et al., 2011; Oellerich et al., 2012; Ng and Tang, 2013; Guarente, 2014; Costa-Machado and Fernandez-Marcos, 2019). Interestingly, controversies regarding the role of sirtuins in various cancers have been acknowledged. For example, SIRT1 has been reported to play a dual role (oncogene/tumor suppressor gene) in different cancers, including liver, lung, breast, pancreas, and colon cancers, through P53-based or other molecular mechanisms (Costa-Machado and Fernandez-Marcos, 2019). SIRT2 can decrease cell glycolysis through pyruvate kinase isoform M2 (PKM2) at lysine 305, which further suppresses tumor cell proliferation in estrogen receptor (ER)-positive breast cancer (Park et al., 2016). However, in triple-negative breast cancer, SIRT2 can promote cancer cell proliferation *via* the deacetylation and stabilization of SLUG (Park et al., 2016). The role of SIRT3 is quite diverse among human malignancies as it serves as an oncogene in lung and colon cancers, but has a tumor-suppressive role in prostate, liver, and breast cancers (Zhang et al., 2012; Desouki et al., 2014; Liu et al., 2014; Quan et al., 2015; Xiong et al., 2017). SIRT4 has been reported to play a role in DNA damage repair, reduce glutamine metabolism *via* ADP-ribosylation of glutamate dehydrogenase, and help in the suppression of cancer proliferation (Jeong et al., 2013). There are also controversial reports on tumor behaviors, such as in cases of breast cancer (Shi et al., 2016; Huang et al., 2017). Questions on the functions of SIRT5 are mainly related to liver cancer. On the one hand, an increased SIRT5 expression level is correlated with poor clinical outcomes *via* the reduction of the E2F1 level (Chang et al., 2017); on the other hand, decreased SIRT5 expression levels can increase the probability of recurrence by maintaining oxidative damage from the peroxisomes (Chen et al., 2018). SIRT6 and SIRT7 are widely investigated in human cancers. Along with other members of the sirtuin family, SIRT6 and SIRT7 also regulate P53, E2F1, PARP, and SMAD4, as well as the functional proteins or miRNAs in cancers with opposing phenotypes (Mao et al., 2011; Sebastián et al., 2012; Kim et al., 2013; Malik et al., 2015; Wu et al., 2015; Zhang S. et al., 2015; Elhanati et al., 2016; Wang et al., 2017).

In summary, the functions of sirtuins in cancer are still under debate. Apparently, most sirtuins may act as tumor suppressors if their expressions are increased. However, sirtuins may act differently under stressful conditions and promote cancer cell proliferation. Thus, sirtuins are alternatives to cancer type and functional context. Currently, the study conflicts are mainly within the same cancer type or pathway. In our study, we decided to investigate the possibility of potential conjoint functions of the sirtuin family members in OC, which may help to further explain the cellular physiology for the different cancer phenotypes.

## Materials and Methods

### Expression Profiles of SIRT Family Members in OC and Normal Tissue by GEPIA

To compare the differential expressions of the sirtuin family with normal tissue, we obtained sirtuin family RNA sequencing (RNA-seq) data from The Cancer Genome Atlas (TCGA) and GTEx using gene expression profiling interactive analysis (GEPIA), which is a web-based tool for RNA-seq analysis (Tang et al., 2017). A heatmap (transcripts per million, TPM) was created to show the difference between OC tissue and normal tissue in the sirtuin family, which was plotted using R 3.5.2 software. The box plots of SIRT1 to SIRT7 were selected to illustrate their differential expression between tumor and normal tissues at transcript levels. In addition, the expressions of SIRT4 and SIRT6 in serous OC at different stages were shown *via* violin plots, and significance was calculated through one-way ANOVA.

### Sirtuin Gene Mutation in TCGA OC Dataset and Methylation Status With mRNA Expression From CCLE

To analyze the mutation patterns of all members in the sirtuin family, the cBioPortal for Cancer Genomics^1^, which is an online tool developed by the Memorial Sloan Kettering Cancer Center and supports an open-access web database for exploring, visualizing, and comprehensively analyzing cancer genomics data from TCGA, was accessed (Cerami et al., 2012; Gao et al., 2013). In our present study, the RNA-seq research cohort “Ovarian Serous Cystadenocarcinoma—TCGA, Firehose Legacy—311 patients” data were used for genomic mutation analysis by querying the gene symbols of the sirtuin family members. A bar plot with proportion was constructed to display the mutation rates among the sirtuin family members. To confirm the association of SIRT4 and SIRT6 messenger RNA (mRNA) expressions with the DNA methylation status in ovarian cancer cell lines, the Cancer Cell Line Encyclopedia (CCLE) database was accessed for all available ovarian cancer cell lines (Ghandi et al., 2019). The DNA methylation status through reduced representation bisulfite sequencing (RRBS) was plotted into a rectangular coordinate system.

### Overall Survival and Progression-Free Survival by Kaplan–Meier Analysis for Serous OC

To evaluate the independent predictive value of ovarian cancer patient survival, the overall survival (OS) and progression-free survival (PFS) reflecting the prognostic value of each member in the sirtuin family were evaluated in the serous OC patient cohort. In addition, the SIRT4/SIRT6 ratio was also calculated as a prognostic marker to be applied in the Kaplan–Meier (KM) plot for OS and PFS. To explore the prognostic value of the sirtuin family, a web tool known as Kaplan–Meier plotter was employed^2^ (Nagy et al., 2018). The KM Plotter contains the OS and PFS data and the data on the expressions of 54,000 genes of over 1,000 OC patients. The data analyzed in the KM Plotter online tool were stratified by median signal expression (high *vs*. low). Hazard ratios and *p*-values (log-rank *p*) are displayed on survival curves. A log-rank *p* < 0.05 was considered statistically significant. The International Cancer Genome Consortium (ICGC) OC research cohort with 82 serous cystadenocarcinoma cases (2016) was used as a validation cohort for OS. In addition, the ratio of SIRT4 to SIRT6 was calculated and used for OS analysis with the best *p*-value to validate the result from the KM Plotter database.

### Representative IHC Staining From the Human Protein Atlas and Clinical Samples

To test the potential use for clinical ovarian cancer samples for SIRT4 and SIRT6 at the practical level, we selected the public database and our sample cohort for validation. The Human Protein Atlas (HPA) is an open-access program aimed at portraying human cells and cancers by analyzing their protein expressions^3^ (Thul et al., 2017). In the present study, representative SIRT4 and SIRT6 immunohistochemical (IHC) staining results were reviewed for normal ovarian and serous OC samples. The staining intensity was re-evaluated by two independent pathologists and designated as “weak,” “moderate,” or “strong” (Yu et al., 2019). Only images scored and designated with consensus were selected as representative images. In addition, we collected 20 ovarian cancer patient samples to perform IHC staining as supporting evidence for SIRT4 and SIRT6. Informed consent was signed by all patients, and the study was approved by the ethics committee of Harbin Medical University. The cancer samples were collected from leftover tumors after diagnosis and processed anonymously under ethics and law from the pathology department. The slides were sectioned at 5 μm and dropped twice in 100% xylene for 5 min and dehydrated through ethanol (100%, 5 min; 100%, 5 min; 90%, 5 min; 80%, 5 min; and 70%, 3 min). The citric acid repair solution was used for 20 min. After cooling, endogenous peroxidase blocking solution was applied to each slide for 10 min and then washed three times with phosphate-buffered saline (PBS) for 5 min. Primary antibody (SIRT4: 1:100; ab231137, Abcam, Cambridge, United Kingdom; SIRT6: 1:100, AF7983, Beyotime, Shanghai, China) was used and incubated at 4°C overnight. The following day, the slides were washed with PBS for 5 min and incubated with the secondary antibody (Maxin, Fujian, China) at room temperature for 60 min. After washing with PBS, diluted DAB (Maxin, Fujian, China) was dropped for 3 min with the counterstain.

### Co-expression of the SIRT4 and SIRT6 Genes Extracted From the OC RNA-Seq Data, DAVID, GO, and KEGG Enrichment Analysis

To explore the underlying molecular functions of SIRT4 and SIRT6, LinkedOmics^4^, which is a portal that contains multi-omics data and clinical information of over 30 TCGA data-supported cancer types including OC, was accessed (Vasaikar et al., 2018). The OC dataset from the University of North Carolina (UNC) RNA-seq data through the HiSeq RNA platform was adopted as the target dataset by running the Firehose_RSEM_log2 pipeline. Pearson’s correlation test was performed. A volcano plot and heatmap with the top 50 positively and negatively correlated genes were extracted to show the Pearson’s correlation coefficients of the SIRT4 and SIRT6 genes through the TCGA ovarian cohort. All positively correlated genes with a Pearson’s correlation score > 0.3 and the negatively correlated genes with a Pearson’s correlation score ≤ 0.3 were selected for GO terms (biological process), Reactome pathways, and Kyoto Encyclopedia of Genes and Genomes (KEGG) pathway analysis using the Database for Annotation, Visualization, and Integrated Discovery (DAVID)^5^, which is a tool for gene annotation with a list of genes of interest (Huang et al., 2008). “*Homo sapiens*” was selected as the background parameter. The top 10 or “If any” enriched GO and KEGG terms are plotted in a bubble chart for SIRT4 or SIRT6.

### SIRT4 and SIRT6 Association With Tumor-Infiltrating Immune Cells

To further explore the association of single gene expressions of SIRT4 and SIRT6 with tumor-infiltrating immune cells, the TIMER 2.0 database was accessed to estimate the immune cell infiltration abundances through multiple immune deconvolution methods (Liu et al., 2020). TCGA-OV, with 303 ovarian cancer patient expression profiles, was used as the analysis landscape. Person’s correlation was calculated to evaluate the association with immune cells.

### ROC Plot for Drug Sensitivity and STRING Database

To verify the potential anticancer drug response according to the expressions of SIRT4 and SIRT6, we analyzed the available data from ROC Plotter. The ROC Plotter is a transcriptome-level open-access database for biomarker validation for independent drug treatment response prediction, which contains 70,632 official gene symbols or aliases of 2,369 OC patients (Fekete and Győrffy, 2019). Samples with relapse-free survival at 6 months (*n* = 1,347) were used. Chemotherapy drugs and targeted therapy (Avastin) were selected. The receiver operating characteristic (ROC) *p*-value and the Mann–Whitney test *p*-value were calculated. Single expressions with SIRT4 and SIRT6 or the combined ratio of SIRT4/SIRT6 was set as a predictor for all the selected drug responses. The protein–protein interaction (PPI) network of SIRT4 and SIRT6 was constructed using the STRING database, version 11^6^, which is a well-known protein function prediction database (Szklarczyk et al., 2019). SIRT4 and SIRT6 were set at an interaction with a combined score of > 0.4 (medium confidence) and no more than 10 interactors. Meanwhile, the network was also classified by the “MCL inflation parameter.”

## Results

### Independent Prognostic Values of the Sirtuin Family Members in OC

**FIGURE 1:**
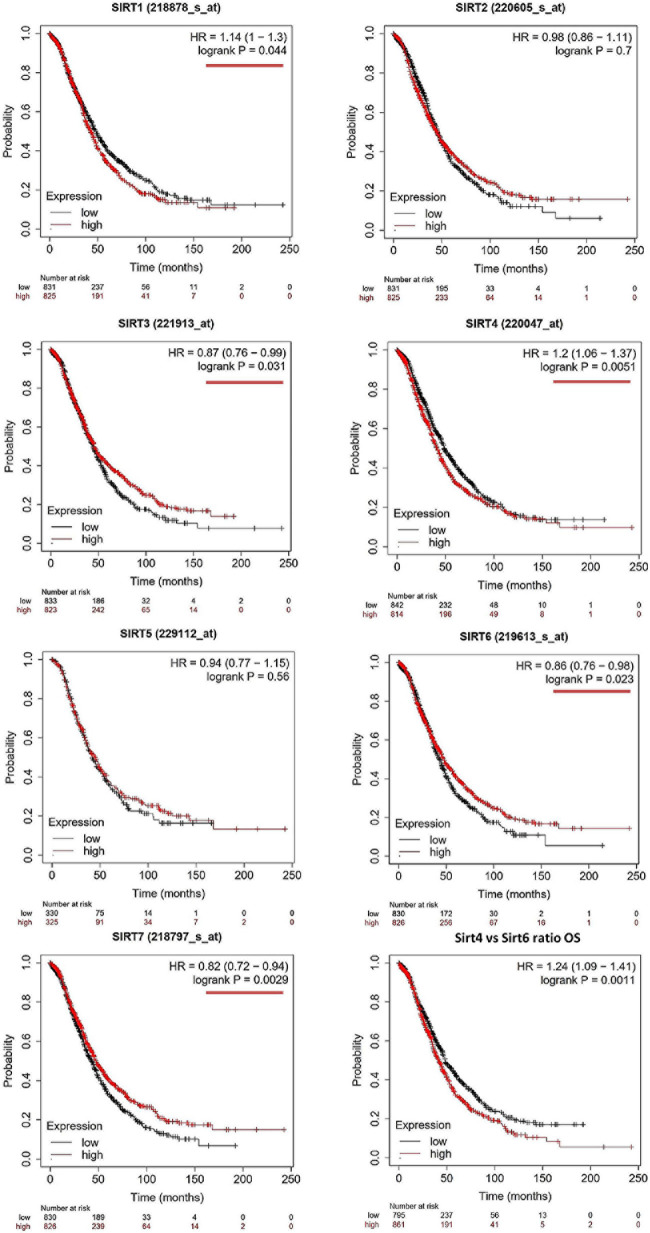
Prognostic values of the sirtuin family members with probe IDs in ovarian cancer patients, as obtained using the Kaplan–Meier (KM) Plotter (*n* = 1,656) for overall survival (OS) analysis. A log-rank *p* < 0.05 was considered statistically significant and is marked with a *red underline*. The median expression level was set as the cutoff for the KM plot. The *red line* indicates high expression and the *blue line* indicates low expression.

**FIGURE 2:**
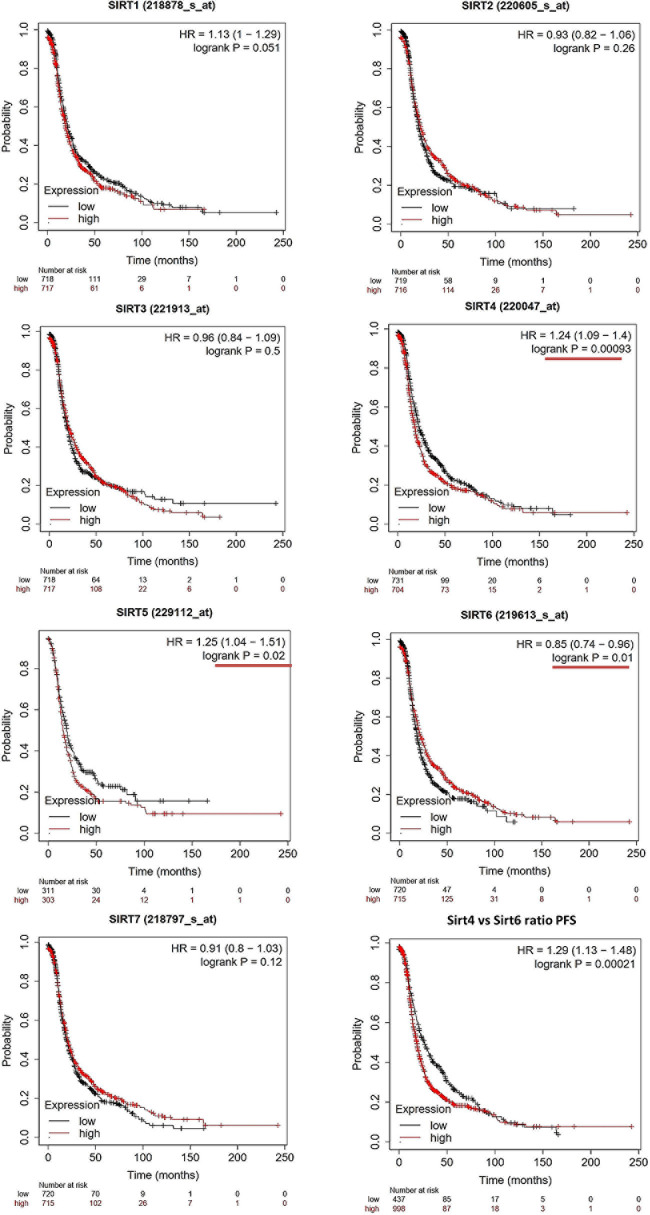
Prognostic values of the sirtuin family members with probe IDs in ovarian cancer patients, as obtained using the Kaplan–Meier (KM) Plotter (*n* = 1,425) for progression-free survival (PFS) analysis. A log-rank *p* < 0.05 was considered statistically significant and is marked with a *red underline*. The median expression level was set as the cutoff for the KM plot. The *red line* indicates high expression and the *blue line* indicates low expression.

The OS and PFS data of ovarian cancer patients from all validation cohorts in the KM Plotter database were used for KM analysis of the sirtuin family members (Figures 1, 2). A total of 1,656 OC cases for OS and 1,435 cases for PFS were divided into high- and low-expression groups at the median signal of each sirtuin family member through the microarray platform.

For OS evaluation (Figure 1), SIRT1 (probe ID: 218878_s_at) showed an unfavorable OS outcome, with a hazard ratio (HR) = 1.14 (1–1.3) and log-rank *p* = 0.044. SIRT2 (probe ID: 220605_s_at) and SIRT5 (probe ID: 229112_at) were not independent prognostic markers for OC, with HR = 0.98 (0.86–1.11), log-rank *p* = 0.7, and HR = 0.94 (0.77–1.15), log-rank *p* = 0.56, respectively. SIRT3 (probe ID: 221913_at) [HR = 0.87 (0.76–0.99), log-rank *p* = 0.031]; SIRT6 (probe ID: 219613_s_at) [HR = 0.86 (0.76–0.98), log-rank *p* = 0.023]; and SIRT7 (probe ID: 218797_s_at) [HR = 0.82 (0.72–0.94), log-rank *p* = 0.0029] were identified as favorable prognostic biomarkers with increasing expression levels. On the contrary, SIRT4 showed a poor OS time when its expression increased, with HR = 1.2 (1.06–1.37) and log-rank *p* = 0.0051.

For PFS prediction (Figure 2), SIRT1 (probe ID: 218878_s_at) displayed a higher expression level with a poor PFS trend, with HR = 1.13 (1–1.29) and log-rank *p* = 0.051. SIRT2 (probe ID: 220605_s_at) and SIRT3 (probe ID: 221913_at) were not useful markers for OC PFS, with HR = 0.93 (0.82–1.06), log-rank *p* = 0.26, and HR = 0.96 (0.84–1.09), log-rank *p* = 0.5, respectively. SIRT4, SIRT5, and SIRT6 were found to be independent biomarkers for OC PFS in this large patient cohort. Specifically, the higher expressions of SIRT4 and SIRT5 indicate a poor PFS outcome, with HR = 1.24 (1.09–1.4), log-rank *p* = 0.00093, and HR = 1.25 (1.04–1.51), log-rank *p* = 0.02, respectively. On the contrary, high SIRT6 levels had a favorable clinical PFS outcome in OC patients, with HR = 0.85 (0.74–0.96) and log-rank *p* = 0.01. SIRT7 did not have any significant impact on PFS in the KM analysis [HR = 0.91 (0.8–1.03) and log-rank *p* = 0.12].

As SIRT4 and SIRT6 were both sensitive as prognostic markers for OS and PFS, the ratio of SIRT4 to SIRT6 was calculated and used for additional analyses of OS and PFS. The results clearly showed that a higher ratio of this group indicated poor survival time in both OS and PFS analyses, with HR = 1.24 (1.09–1.41), log-rank *p* = 0.0011, and HR = 1.29 (1.13–1.48), log-rank *p* = 2.1E−4, respectively (Figures 1, 2, bottom right panel). To validate the KM Plotter cohort, the results for OS from the ICGC (total *n* = 82) showed the following values for the high- *vs*. low-risk group: SIRT4: HR = 1.61 (0.99–2.63), log-rank *p* = 0.05568 (Supplementary Figure 1A); SIRT6: HR = 0.86 (0.53–1.39), log-rank *p* = 0.5297 (Supplementary Figure 1B); and SIRT4/SIRT6 ratio: HR = 1.71 (1.05–2.79), log-rank *p* = 0.03266 (Supplementary Figure 1C).

### Sirtuin Family Mutations, Expressions, and Association With OC Stages

**FIGURE 3:**
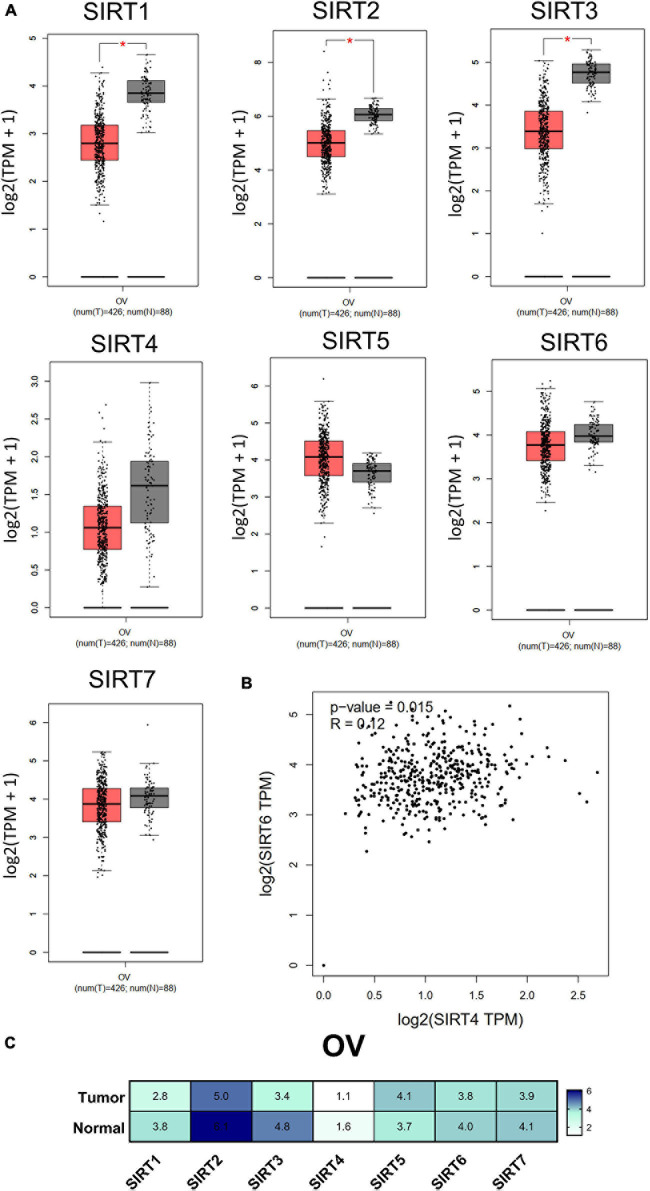
Differential expressions of the sirtuin family members in ovarian cancer and normal ovarian tissues. **(A)** Comparison of normalized sirtuin family member expression levels in tumor samples *vs*. those in normal tissues. The cancer abbreviation name is shown according to The Cancer Genome Atlas (TCGA) study abbreviation (*OV*, ovarian cancer). *p < 0.05 was considered statistically significant. **(B)** Dot plot of SIRT4 and SIRT6 correlations calculated by Pearson’s correlation test from TCGA-OV. **(C)** Heatmap of the expressions of the sirtuin family members by log_2_(TPM + 1) for log-scale (*number in the cell*).

Transcriptional expression analysis of the RNA-seq data obtained from TCGA database was performed using GEPIA. We found that there were three members (SIRT1, SIRT2, and SIRT3) with differential expressions among the OC (*n* = 426) and normal ovary (*n* = 88) tissue samples. The remaining members (SIRT4, SIRT5, SIRT6, and SIRT7) in the sirtuin family did not show statistical differences between the normal ovary and OC tissue samples (Figure 3A). The sirtuin family member with the highest expression in OC was SIRT2 (5.0 TPM), while SIRT4 had the lowest expression (1.1 TPM); these data were plotted on a heatmap (Figure 3C). As SIRT4 and SIRT6 were both independent prognostic markers for OS and PFS, we further determined the correlation between SIRT4 and SIRT6 with Pearson’s correlation analysis (*p* = 0.015, *R* = 0.12) (Figure 3B).

**FIGURE 4:**
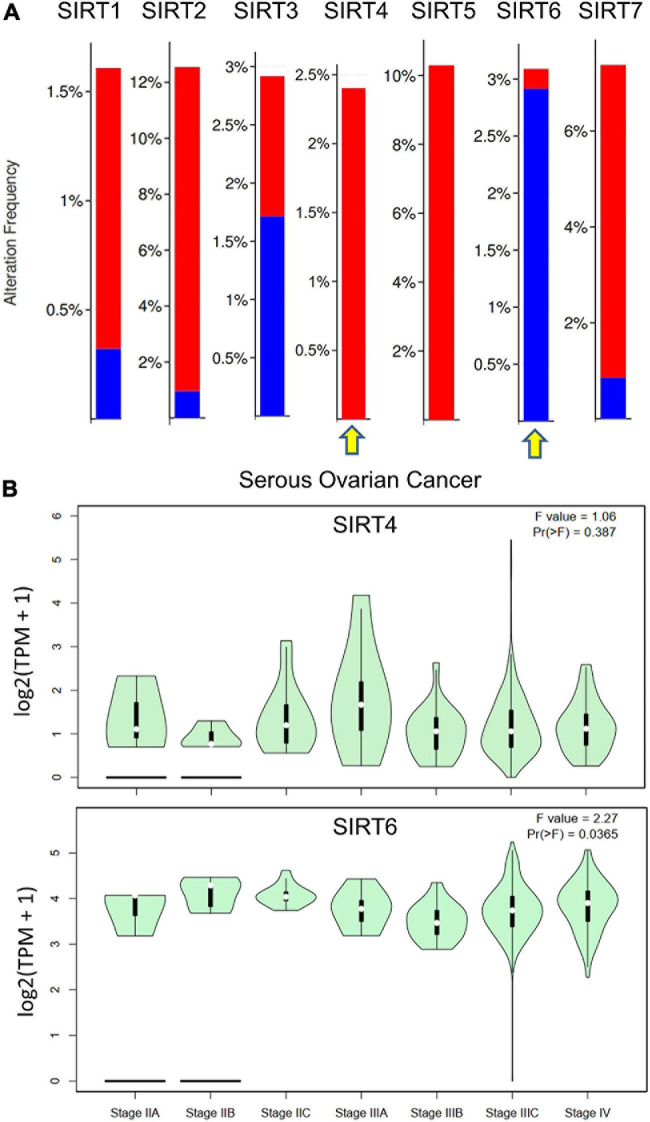
Gene mutation rates of selected sirtuin family members and their association with clinical stages. **(A)** Bar plot of the mutation rate (proportions) in The Cancer Genome Atlas ovarian cancer (TCGA-OV) data cohort. *Red bar* indicates copy number variation (CNV) amplification and *blue bar* indicates deep deletion. *Yellow arrow* indicates the selected members for downstream analysis. **(B)** Violin plots of the relationship between SIRT4/SIRT6 expressions and the tumor stages of patients from TCGA-OV.

In addition, we accessed the mutation data from TCGA dataset [Ovarian Serous Cystadenocarcinoma (TCGA, Firehose Legacy), 594 patients] for the sirtuin family. The alteration frequency is plotted as a bar chart. The analysis revealed that the *SIRT1* gene was altered in 1.89% of 583 cases [amplification = 1.54% (9 cases), deep deletion = 0.34% (2 cases)]. The *SIRT2* gene was altered in 12.18% of 583 cases [amplification = 11.32% (66 cases), deep deletion = 0.86% (5 cases)]. The SIRT3 gene was altered in 2.92% of 583 cases [(amplification = 1.2% (7 cases), deep deletion = 1.72% (10 cases)]. The *SIRT4* gene was altered in 2.4% of 583 cases [(amplification = 2.4% (14 cases)]. The SIRT5 gene was altered in 10.29% of 583 cases [(amplification, 10.29% (60 cases)]. The *SIRT6* gene was altered in 3.09% of 583 cases [(amplification = 0.17% (1 case), deep deletion = 2.92% (17 cases)]. The *SIRT7* gene was altered in 7.38% of 583 cases [(amplification = 6.52% (38 cases), deep deletion = 0.86% (5 cases)]. The opposite gene mutation patterns were found in SIRT4 and SIRT6 (Figure 4A). Moreover, the major stage analysis for SIRT4 and SIRT6 in OC showed negative results, as there were no significant differences in their average expressions among the OC stages. The *F*-test values are indicated in the violin plot (Figure 4B).

For the analysis of the gene expression association with their promoter DNA methylation in ovarian cancer cell lines, our results showed that SIRT4 had significantly fewer methylations than does SIRT6, but there was one cell line that had up to 0.2 correlation more than others (Supplementary Figure 2B). Notably, many ovarian cancer cell lines did not have any SIRT4 promoter methylation, where SIRT6 showed the opposite manner (Supplementary Figure 2C). In comparing the mRNA RNA-seq expressions, SIRT6 methylation gained a decreased mRNA expression pattern.

### The ROC Plot for OC Chemotherapy and Targeted Therapy in the Prediction of Relapse-Free Survival Time at 6 Months

The total number of cases are as follows: for Plantin was 1,209 (non-responders = 114, responders = 1,095), for docetaxel was 97 (non-responders = 5, responders = 92), for paclitaxel was 208 (non-responders = 20, responders = 188), for gemcitabine was 126 (non-responders = 7, responders = 119), for Topotecan was 118 (non-responders = 5, responders = 113), and for Avastin was 50 (non-responders = 3, responders = 47).

**FIGURE 5:**
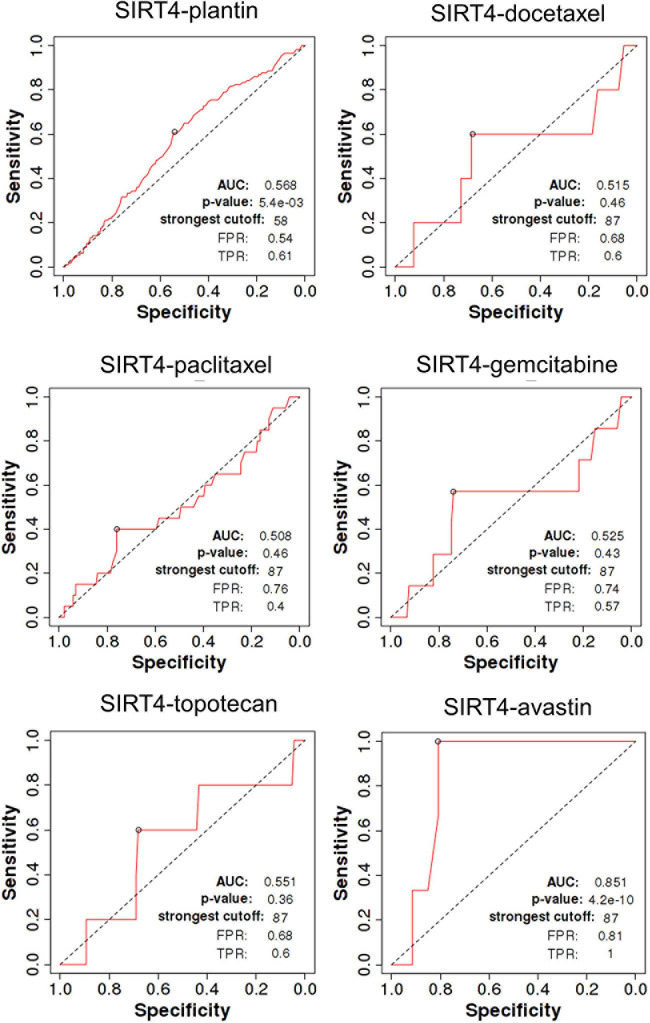
Receiver operating characteristic (ROC) curve of SIRT4 in ovarian cancer patients under the indicated drug treatment for relapse-free survival time at 6 months. AUC, area under curve; FPR, false positive rate; TPR, true positive rate.

**FIGURE 6:**
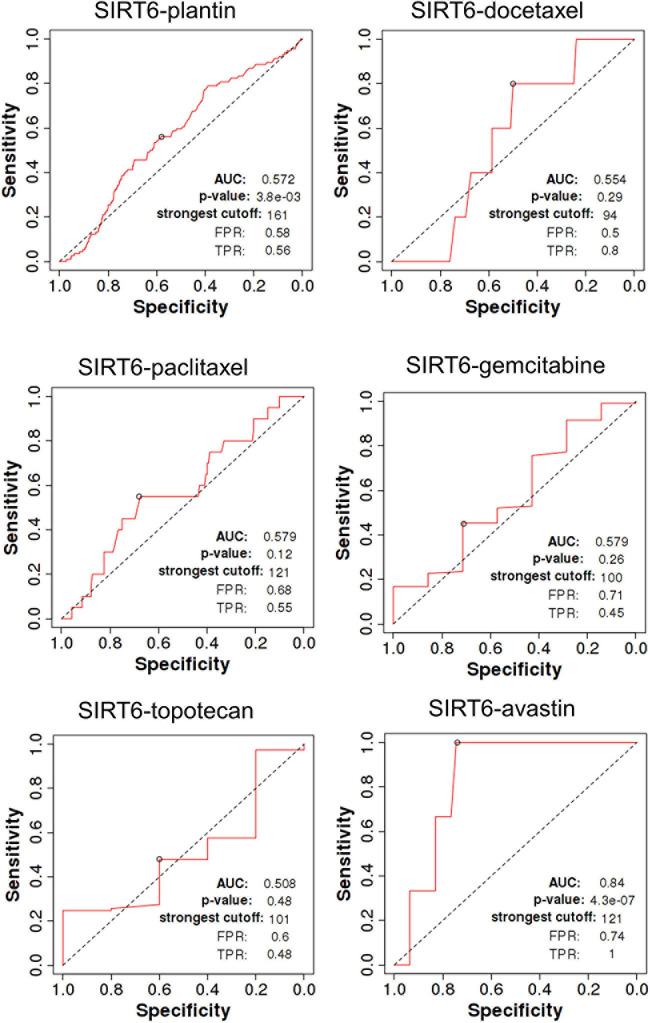
Receiver operating characteristic (ROC) curve of SIRT6 in ovarian cancer patients under the indicated drug treatment for relapse-free survival time at 6 months. AUC, area under curve; FPR, false positive rate; TPR, true positive rate.

**FIGURE 7:**
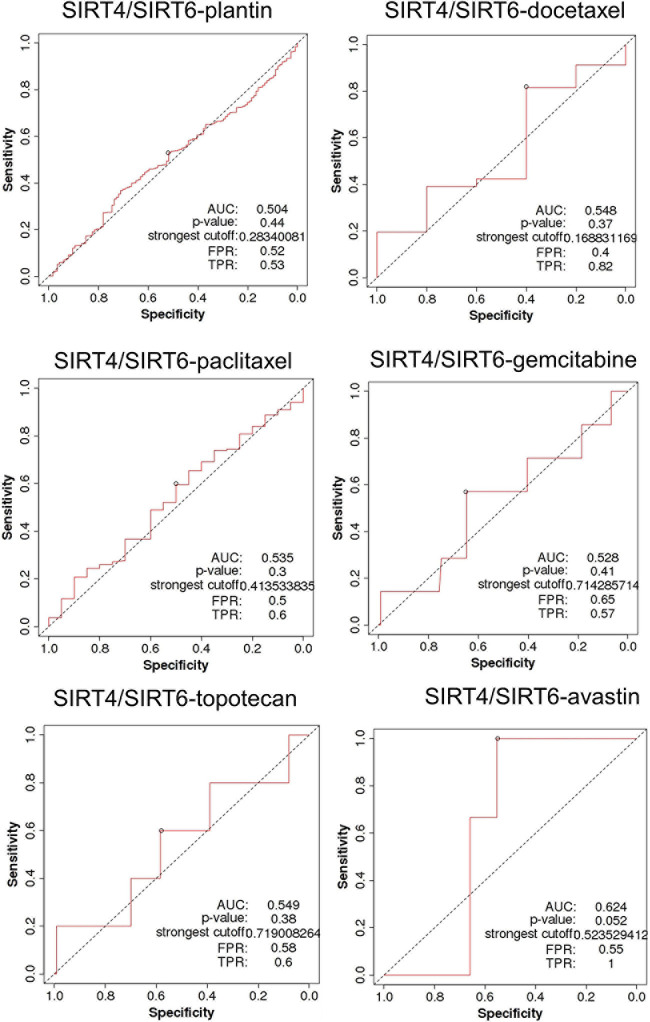
Receiver operating characteristic (ROC) curve of the SIRT4/SIRT6 ratio in ovarian cancer patients under the indicated drug treatment for relapse-free survival time at 6 months. AUC, area under curve; FPR, false positive rate; TPR, true positive rate.

SIRT4 is a relatively sensitive biomarker for predicting the effect of Plantin and Avastin treatments on OC patients [area under the curve (AUC) = 0.588, *p* = 5.4E−03, false positive rate (FPR) = 0.54; AUC = 0.811, *p* = 2.9E−06, FPR = 0.77, respectively]. For the other drugs—docetaxel, paclitaxel, gemcitabine, and topotecan—SIRT4 was not identified as a predictive biomarker (Figure 5). Meanwhile, SIRT6 is also a sensitive marker for Plantin and Avastin, but not for the other drugs included in this analysis (Figure 6). In addition, the combined prediction model using the ratio of SIRT4/SIRT6 for drug response prediction showed AUC values of 0.504, 0.548, 0.535, 0.528, and 0.549 for Plantin, docetaxel, paclitaxel, gemcitabine, and topotecan, respectively. For Avastin, the AUC increased to 0.624, with *p* = 0.052 and FPR = 0.55 (Figure 7).

### Identification of SIRT4- and SIRT6-Correlated Genes in OC From TCGA RNA-Seq Dataset

**FIGURE 8:**
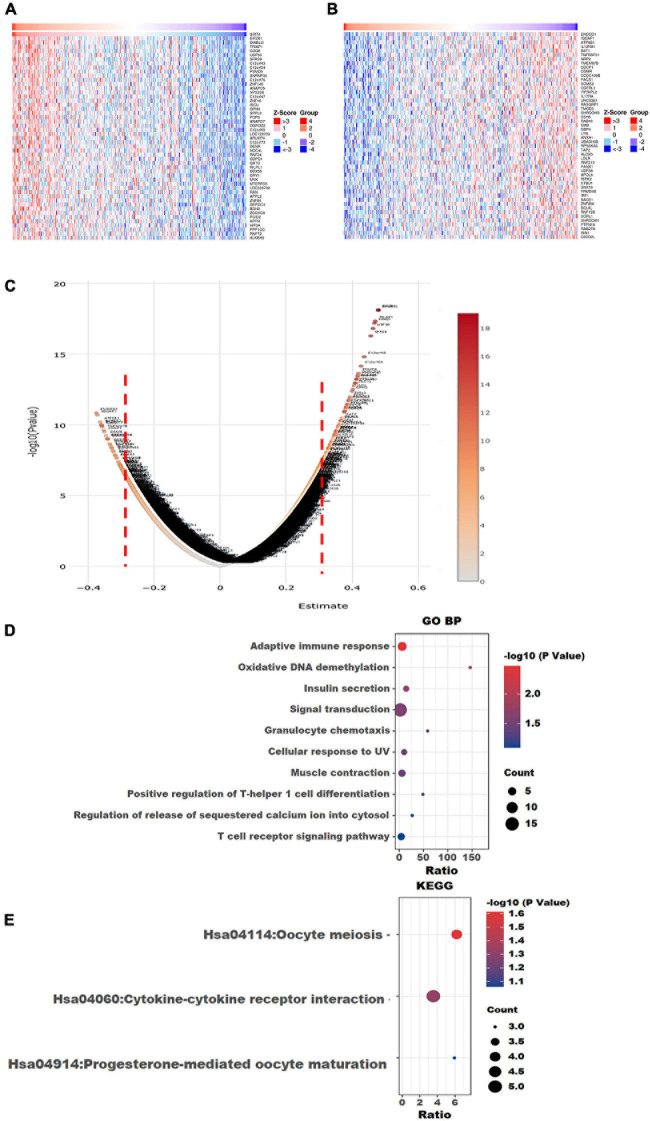
Visualization of Gene Ontology (GO) and Kyoto Encyclopedia of Genes and Genomes (KEGG) enrichment analyses for the SIRT4-correlated genes from The Cancer Genome Atlas ovarian cancer (TCGA-OV). **(A)** Heatmap of the top 50 positively correlated genes of SIRT4 from TCGA-OV after normalization. **(B)** Heatmap of the top 50 negatively correlated genes of SIRT4 from TCGA-OV after normalization. *Red* indicates cases with higher expression and *blue* indicates cases with lower expression (*Z*-score). **(C)** Volcano plot of all correlated genes of SIRT4 in TCGA-OV, as determined using Pearson’s correlation test. *X*-axis is the correlation coefficient. *Red dotted line* indicates the cutoff value for GO and KEGG analyses. **(D)** Top 10 terms of GO BP (biological process) enrichment analysis of the selected genes with Pearson’s correlation > | 0.3| for SIRT4. **(E)** KEGG enrichment analysis using genes as per Pearson’s correlation test. The *p*-value was calculated and sorted with −log_1__0_(*P*). *Dark red* indicates the lowest *p*-value.

**FIGURE 9:**
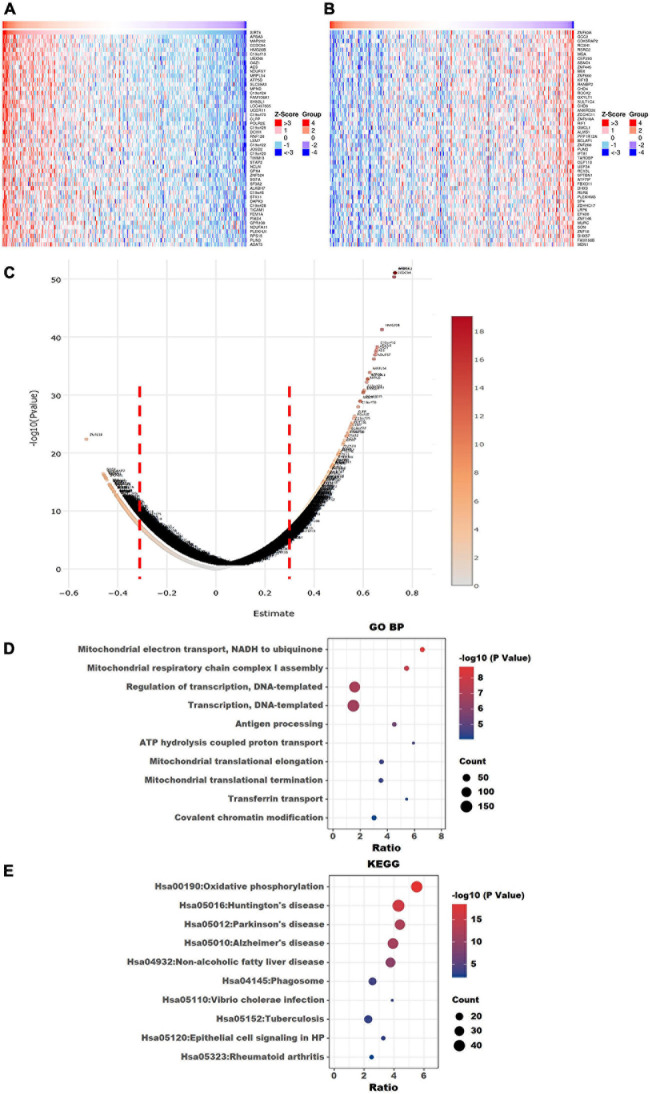
Visualization of Gene Ontology (GO) and Kyoto Encyclopedia of Genes and Genomes (KEGG) enrichment analyses for SIRT6 correlated genes from The Cancer Genome Atlas ovarian cancer (TCGA-OV). **(A)** Heatmap of the top 50 positively correlated genes of SIRT6 from TCGA-OV after normalization. **(B)** Heatmap of the top 50 negatively correlated genes of SIRT6 from TCGA-OV after normalization. *Red* indicates cases with higher expression and *blue* indicates cases with lower expression (*Z*-score). **(C)** Volcano plot of all correlated genes of SIRT6 in TCGA-OV, as determined using Pearson’s correlation test. *X*-axis is the correlation coefficient. *Red dotted line* indicates the cutoff value for GO and KEGG analyses. **(D)** Top 10 terms of GP BP (biological process) enrichment analysis of the selected genes with Pearson’s correlation > | 0.3| for SIRT6. **(E)** KEGG enrichment analysis using genes as per Pearson’s correlation test. The *p*-value was calculated and sorted with −log_1__0_(*P*). *Dark red* indicates the lowest *p*-value.

The top 50 positively correlated and the top 50 negatively correlated genes with SIRT4 and SIRT6 were extracted from TCGA cohort and plotted as heatmaps, respectively (Figures 8A,B, 9A,B). All the genes are displayed in a volcano plot to show the distribution of the positively and negatively correlated genes and the cutoff values for downstream GO and KEGG analyses (Figures 8C, 9C). The top 10 positively and negatively correlated genes of SIRT4 and SIRT6 are summarized in Table 1.

**TABLE 1:** Top 10 SIRT4-positive correlated genes.

**Gene symbol**	**Pearson’s correlation**	***p*-value**	**FDR**
*EIF2B1*	0.479826	7.48E−19	5.37E−15
*DIABLO*	0.479436	8.05E−19	5.37E−15
*TRIAP1*	0.470034	4.65E−18	2.33E−14
*COQ5*	0.46814	6.58E−18	2.63E−14
*USP30*	0.463398	1.55E−17	5.18E−14
*SFRS9*	0.456602	5.19E−17	1.49E−13
*C12orf43*	0.436587	1.56E−15	3.92E−12
*C12orf24*	0.427271	7.08E−15	1.58E−11
*PSMD9*	0.419768	2.31E−14	4.63E−11
*SNRNP35*	0.417502	3.28E−14	5.98E−11
**Top 10 SIRT4−negative correlated genes**			
*ENDOD1*	–0.37533	1.43E−11	8.95E−09
*IQGAP1*	–0.37265	2.04E−11	1.24E−08
*ATP8B1*	–0.36379	6.52E−11	3.53E−08
*IL13RA1*	–0.36066	9.73E−11	5.00E−08
*BST1*	–0.3602	1.03E−10	5.06E−08
*TNFRSF21*	–0.35885	1.22E−10	5.84E−08
*NRP2*	–0.35781	1.40E−10	6.36E−08
*TMEM87B*	–0.3536	2.37E−10	1.01E−07
*CDCP1*	–0.35118	3.19E−10	1.23E−07
*OSMR*	–0.346	6.01E−10	2.08E−07
**Top 10 SIRT6-positive correlated genes**			
*APBA3*	0.730837	7.45E−52	5.95E−48
*MAP2K2*	0.730457	8.92E−52	5.95E−48
*CCDC94*	0.727196	4.12E−51	2.06E−47
*HMG20B*	0.677313	5.08E−42	2.04E−38
*C19orf10*	0.658444	4.93E−39	1.65E−35
*UBXN6*	0.655181	1.54E−38	4.41E−35
*OAZ1*	0.652491	3.90E−38	9.77E−35
*AES*	0.649243	1.18E−37	2.63E−34
*NDUFS7*	0.64445	5.93E−37	1.19E−33
*MRPL54*	0.627718	1.32E−34	2.41E−31
**Top 10 Sirt6−negative correlated genes**			
*ZNF638*	–0.5274	4.29E−23	2.77E−20
*GCC2*	–0.45866	3.62E−17	1.29E−14
*CDK5RAP2*	–0.45539	6.43E−17	2.26E−14
*RC3H1*	–0.45406	8.10E−17	2.75E−14
*RSRC2*	–0.45179	1.20E−16	4.01E−14
*MGA*	–0.44951	1.78E−16	5.66E−14
*CEP290*	–0.4489	1.98E−16	6.19E−14
*SBNO1*	–0.44666	2.90E−16	8.79E−14
*ZNF445*	–0.44362	4.85E−16	1.45E−13
*BBX*	–0.43353	2.58E−15	6.71E−13

*FDR, false discovery rate*

### GO and KEGG Enrichment Analyses and Immune Cell Infiltration Analyses for SIRT4 and SIRT6

To understand the comprehensive biological function network underlying the positively and negatively correlated genes with SIRT4 and SIRT6, GO biological function (GO BP) and KEGG pathway enrichment analyses were performed using DAVID. The top 10 GO terms and KEGG pathways (if any) enriched by the correlated genes > |0.3| are shown in Figures 8D,E, 9D,E. The detailed results of the top enriched GO terms and KEGG pathways are listed in Table 2. For the immune cell infiltration analyses, the TIMER2.0 estimation model was applied and the results shown in Table 3. There was no significant correlation between SIRT4 or SIRT6 expression with T or B cell infiltration.

**TABLE 2:** Top Gene Ontology (GO) terms and Kyoto Encyclopedia of Genes and Genomes (KEGG) pathways for SIRT4 and SIRT6.

**Term**	**Count**	**Ratio**	***p*-value**
**Top 20 GO BP terms for SIRT4**			
GO:0002250—adaptive immune response	6	5.919624	0.00338
GO:0035511—oxidative DNA demethylation	2	146.0174	0.013532
GO:0030073—insulin secretion	3	14.13072	0.018687
GO:0007165—signal transduction	15	1.88653	0.025521
GO:0071621—granulocyte chemotaxis	2	58.40696	0.033491
GO:0034644—cellular response to UV	3	9.955731	0.035914
GO:0006936—muscle contraction	4	5.458594	0.036362
GO:0045627—positive regulation of T-helper 1 cell differentiation	2	48.67246	0.040054
GO:0051279—regulation of release of sequestered calcium ion into cytosol	2	26.54862	0.072215
GO:0050852—T cell receptor signaling pathway	4	3.946416	0.079732
GO:0007265—Ras protein signal transduction	3	6.257888	0.081927
GO:0051436—negative regulation of UPLA involved in mitotic cell cycle	3	6.169749	0.08394
GO:0001817—regulation of cytokine production	2	22.46421	0.084778
GO:1902166—negative regulation of intrinsic apoptotic signaling pathway by p53 class mediator	2	22.46421	0.084778
GO:0050709—negative regulation of protein secretion	2	22.46421	0.084778
GO:0007030—Golgi organization	3	5.919624	0.09007
GO:0030889—negative regulation of B cell proliferation	2	20.85963	0.090997
GO:0032743—positive regulation of interleukin-2 production	2	20.85963	0.090997
GO:0051437—positive regulation of UPLA involved in regulation of mitotic cell cycle transition	3	5.763844	0.094228
**Top KEGG pathways for SIRT4**			
hsa04114: Oocyte meiosis	4	6.197297	0.02453
hsa04060: Cytokine–cytokine receptor interaction	5	3.53858	0.047526
hsa04914: Progesterone-mediated oocyte maturation	3	5.930172	0.086734
**Top 20 GO BP terms for SIRT6**			
GO:0032981—mitochondrial respiratory chain complex I assembly	18	5.421146	1.61E−08
GO:0006355—regulation of transcription, DNA-templated	126	1.589578	1.37E−07
GO:0006351—transcription, DNA-templated	154	1.494628	2.11E−07
GO:0002479—antigen processing and presentation of exogenous peptide antigen *via* MHC class I, TAP-dependent	15	4.517622	3.79E−06
GO:0015991—ATP hydrolysis coupled proton transport	10	5.929379	2.79E−05
GO:0070125—mitochondrial translational elongation	16	3.571579	3.33E−05
GO:0070126—mitochondrial translational termination	16	3.530049	3.84E−05
GO:0033572—transferrin transport	10	5.421146	6.09E−05
GO:0016569—covalent chromatin modification	18	3.022409	8.46E−05
GO:0042384—cilium assembly	19	2.907308	8.52E−05
GO:0016032—viral process	32	2.030663	2.51E−04
GO:0000209—protein polyubiquitination	23	2.371751	2.77E−04
GO:0090383—phagosome acidification	8	5.621929	3.84E−04
GO:0042776—mitochondrial ATP synthesis coupled proton transport	7	6.32467	5.74E−04
GO:0006754—ATP biosynthetic process	8	5.23421	6.16E−04
GO:0008286—insulin receptor signaling pathway	13	3.162335	7.38E−04
GO:0060271—cilium morphogenesis	18	2.511266	7.96E−04
GO:0019886—antigen processing and presentation of exogenous peptide antigen *via* MHC class II	14	2.88735	0.001028
GO:0097190—apoptotic signaling pathway	12	3.206875	0.001142
**Top KEGG pathways for SIRT6**			
hsa00190: Oxidative phosphorylation	40	5.516992	3.94E−19
hsa05016: Huntington’s disease	45	4.299375	5.93E−17
hsa05012: Parkinson’s disease	34	4.392225	4.39E−13
hsa05010: Alzheimer’s disease	36	3.930857	2.56E−12
hsa04932: Non-alcoholic fatty liver disease	31	3.765987	3.48E−10
hsa04145: Phagosome	21	2.56816	1.62E−04
hsa05110: *Vibrio cholerae* infection	11	3.880462	4.06E−04
hsa05152: Tuberculosis	22	2.280045	5.71E−04
hsa05120: Epithelial cell signaling in HP	12	3.285493	8.67E−04
hsa05323: Rheumatoid arthritis	12	2.501455	0.007791
hsa00240: Pyrimidine metabolism	13	2.361109	0.008321
hsa03050: Proteasome	8	3.335273	0.008894
hsa05020: Prion diseases	7	3.776706	0.009131
hsa04966: Collecting duct acid secretion	6	4.076444	0.013855
hsa04142: Lysosome	14	2.122446	0.013906
hsa05132: *Salmonella* infection	11	2.431133	0.014002
hsa04064: NF-kappa B signaling pathway	11	2.319356	0.019028
hsa05133: Pertussis	10	2.445867	0.019728
hsa04260: Cardiac muscle contraction	10	2.445867	0.019728
hsa04721: Synaptic vesicle cycle	9	2.620571	0.019968

**TABLE 3:** Immune cell infiltration analyses with the TIMER2.0 estimation model.

**Immune estimation algorithms**	**OV-SIRT4**	**OV-SIRT6**
B cell XCELL	–0.04	0.127
B cell MCP-COUNTER	0.132	–0.02
B cell memory CIBERSORT	–0.03	0.066
B cell memory CIBERSORT-ABS	–0.03	0.073
B cell memory XCELL	0.114	0.091
B cell naive CIBERSORT	0.008	–0.06
B cell naive CIBERSORT-ABS	0.012	–0.01
B cell naive XCELL	0.094	–0.06
B cell plasma CIBERSORT	0.027	–0.1
B cell plasma CIBERSORT-ABS	0.042	–0.03
B cell plasma XCELL	0.079	0.044
Class-switched memory B cell XCELL	–0.09	0.111
T cell CD8^+^ TIMER	0.022	0.017
T cell CD8^+^ EPIC	–0.033	–0.154
T cell CD8^+^ MCPCOUNTER	–0.023	0.122
T cell CD8^+^ CIBERSORT	–0.016	0.061
T cell CD8^+^ CIBERSORT-ABS	0.006	0.152
T cell CD8^+^ QUANTISEQ	–0.058	0.134
T cell CD8^+^ XCELL	0.103	–0.023
T cell CD8^+^ naive_XCELL	0.294	–0.091
T cell CD8^+^ central memory_XCELL	–0.144	0.034
T cell CD8^+^ effector memory_XCELL	0.033	–0.056
T cell CD4^+^ EPIC	–0.045	–0.065
T cell CD4^+^ TIMER	0.013	0.134
T cell CD4^+^ (non-regulatory) QUANTISEQ	–0.104	–0.024
T cell CD4^+^ (non-regulatory) XCELL	0.006	–0.02
T cell CD4^+^ naive CIBERSORT	0.128	–0.09
T cell CD4^+^ naive CIBERSORT-ABS	0.128	–0.09
T cell CD4^+^ naive XCELL	0.064	0.074
T cell CD4^+^ memory XCELL	–0.083	–0.152
T cell CD4^+^ central memory XCELL	0.135	0.026
T cell CD4^+^ effector memory XCELL	–0.087	0.095
T cell CD4^+^ memory activated CIBERSORT	–0.075	–0.001
T cell CD4^+^ memory activated CIBERSORT-ABS	–0.075	–0.002
T cell CD4^+^ memory resting CIBERSORT	–0.045	–0.074
T cell CD4^+^ memory resting CIBERSORT-ABS	–0.022	0.052
T cell CD4^+^ Th1 XCELL	–0.01	0.191

### Representative IHC Staining of SIRT4 and SIRT6 in Clinical Samples

**FIGURE 10:**
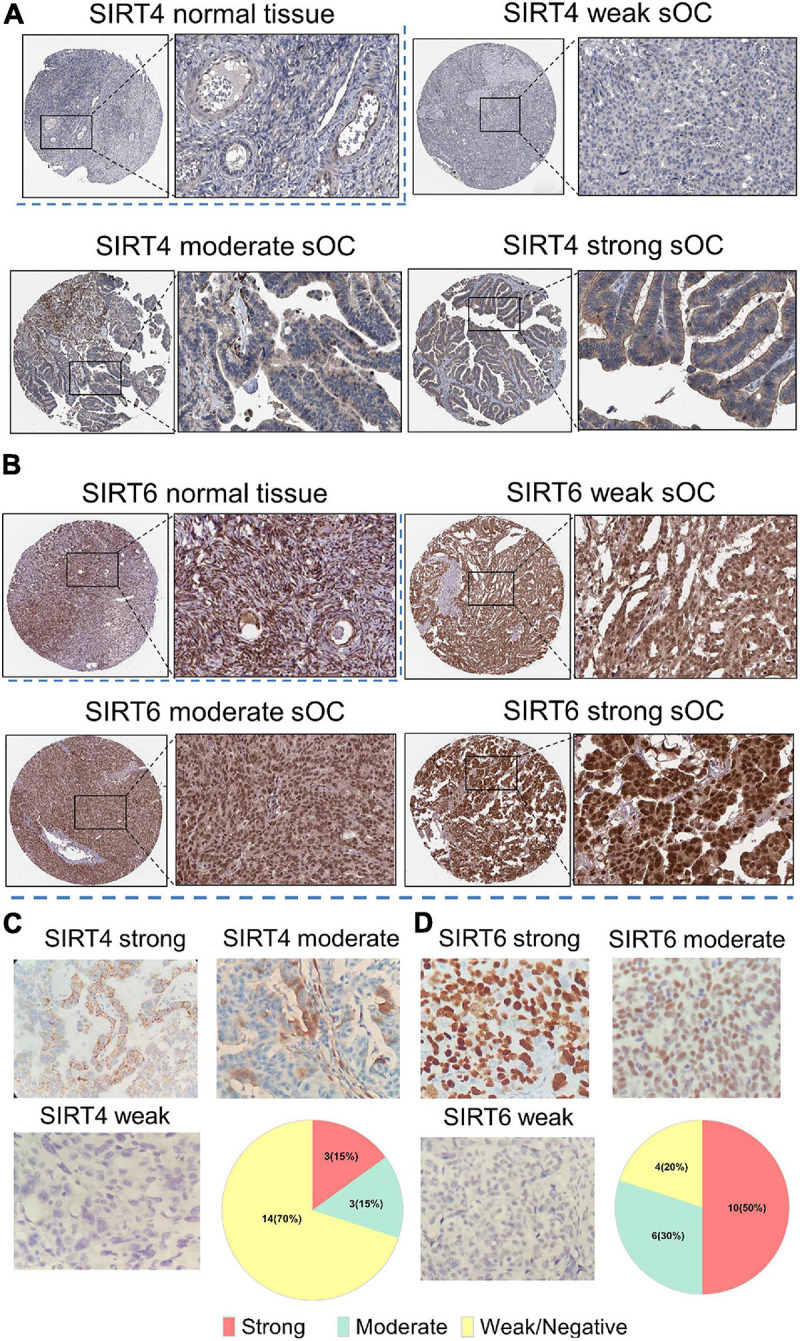
Immunohistochemistry of SIRT4 and SIRT6 in samples from normal ovaries and serous ovarian cancer patients (sOC). **(A,B)** Image in the *upper left panel* is for the SIRT4/SIRT6 staining intensity of normal ovary tissues. Images in the rest of the panel are representative pictures of SIRT4/SIRT6 staining with an intensity from weak to strong. *Black rectangle* indicates the zoomed-in zone in the image. **(C)** Panel of SIRT4 immunohistochemical (IHC) staining pattern. *Upper left*, strong; *upper right*, moderate; *lower left*, weak or negative; *lower right*, pie chart of the indicated case numbers (total *n* = 20). **(D)** Panel of SIRT6 IHC staining pattern. *Upper left*, strong; *upper right*, moderate; *lower left*, weak or negative; *lower right*, pie chart of the indicated case numbers (total *n* = 20).

We further obtained SIRT4 and SIRT6 IHC expression patterns from the HPA. For SIRT4, the normal ovary tissue showed very weak staining in the cytoplasm (Figure 10A). In the OC sample, the representative staining of SIRT4 showed an almost negative and a weak staining pattern. The moderate and strong patterns showed dark/intense brown intensity in the cytoplasm and could not be easily distinguished. For SIRT6, the normal ovary displayed dark brown staining in the nucleus. In serous OC samples, the intensity of the brown staining of the nucleus increased from relatively weak to strong (Figure 10B). To validate the IHC staining expression patterns, 20 ovarian cancer clinical resection samples were further stained with SIRT4 and SIRT6. The staining pattern for SIRT4 was majorly shown in the cytoplasm and membrane. Only three cases (15%) were stained strongly. Fourteen cases (70%) were very weak or negative for SIRT4 (Figure 10C). The IHC staining pattern for SIRT6 was consistent with the online database, which is strongly at the nucleus. Ten cases (50%) were at a strong signal, whereas four cases (20%) showed a negative or a weak staining pattern (Figure 10D).

### SIRT4 and SIRT6 Protein–Protein Function Prediction Through MCL Clustering

The PPI network for the interaction between SIRT4 and SIRT6 was constructed using the parameters described in section “Materials and Methods.” We found 12 nodes and 41 edges, with an average node degree of 6.83. Moreover, MCL clustering showed that SIRT6 may have a function that is contradictory to SIRT4, with their core interacting proteins under an average local clustering coefficient of 0.879. The PPI enrichment *p*-value was estimated to be 1.3E−06 (Supplementary Figure 2A).

## Discussion

As traditional chemotherapy is frequently associated with drug resistance, the challenge for the successful treatment of OC is to identify the patient cohort that can benefit from targeted therapy. Thus, there is an urgent requirement for effective and predictable biomarkers for OC (Custodio et al., 2012). Evidence implies that the sirtuin family members play important roles in cancer development through their regulation of metabolic functions. According to controversial reports on the different tumor types or subtypes, the sirtuin family members exhibit complex and dual roles in various human cancers, including OC.

As we intended to explore the role of each member in the sirtuin family for its potential life span prediction value and drug effectiveness with regard to OC patients, comprehensive bioinformatics-based analyses were performed. Previous reports on SIRT1 have shown its role not only in chemoresistance but also in OC development through known classical molecular mechanisms such as *BRCA1* interaction (Li et al., 2014). With regard to its role in predicting clinical outcomes, SIRT1 exhibits certain conflicts within different research cohorts. For example, a higher IHC staining score was correlated to a poor OS outcome in a cohort of 68 OC patients (*p* = 0.038), and this finding was supported by the study results of Shuang et al. (2015) and Mvunta et al. (2017). However, the opposite result was reported by Wang et al. (2008), wherein, through a comprehensive study involving transgenic mice, SIRT1 was identified as a tumor suppressor). In the present study, SIRT1 was related to poor clinical OS time and was not correlated with PFS time.

According to our results, SIRT1, SIRT2, and SIRT3 were all downregulated in OC tissues compared to those in normal ovarian tissues. However, none of the three markers were sensitive to PFS. For OS time, our results are not similar to those reported by Teng and Zheng (2017) as their study found that SIRT2, a target gene of miR1908, was found to be associated with poor prognosis in OC. Li et al. (2019) have also shown that SIRT3 is an independent favorable prognostic factor for OS in serous OC. SIRT3 has also been found to be crucial for anchorage-independent survival and metastasis of OC cells, and both these processes are known to be critical for OC disease progression (Kim et al., 2019). More importantly, the SIRT3/hypoxia-inducible factor (HIF) axis is significantly involved in the Warburg effect observed in OC cells under treatment with ABT737 (Dong et al., 2020).

SIRT5 is not as frequently studied as the other members of OC. A report based on three classic OC cell lines has revealed its role in promoting cisplatin resistance in an ROS-dependent manner. NRF2/HO-1 is the downstream target of this regulation axis (Sun et al., 2019a). Meanwhile, the amplification mutation rate of SIRT5 is relatively higher than that of the other members of the sirtuin family, and this is a matter of interest. Li et al. (2019) have also pointed out that, in addition to SIRT3, SIRT5 and SIRT7 are associated with better clinical outcomes for OS, with data from TCGA and GSE9891 datasets. In our study, SIRT5 was found to be a potential biomarker for PFS time, but was not related to OS time. Furthermore, SIRT7 was found to be highly expressed in OC cell lines as compared to its expression in ovary surface epithelium cells and to exhibit an oncogenic potential in OC cells (Wang et al., 2015). In addition, the chemoresistance of OC cells could be inhibited by the upregulation of SIRT7 (Aljada et al., 2014).

Notably, SIRT4 and SIRT6 both had significant prognostic values in OS and PFS in comparison to those of the other members of the sirtuin family. Sun et al. (2019b) and He et al. (2020) also analyzed the sirtuin family members by using comprehensive bioinformatics tools, including KM plots, with different results for SIRT4 and SIRT6. However, their research had certain limitations and shortcomings with regard to the methods and result interpretation or analysis of the data from a different biological point of view.

SIRT4 is generally believed to be a tumor suppressor in many human malignancies as the tumor loses its expression. The protein expression level of SIRT4 was also evaluated by using IHC in a study by Fu et al., which was consistent with the mRNA expression level of SIRT4 (Fu et al., 2016). Our results using TCGA RNA-seq and IHC supported the same expression pattern of SIRT4 for OC and normal ovary tissues. However, it remains a question whether the low expression of SIRT4 protein affects the IHC result interpretation with non-specific staining. The use of a transgenic mouse model for cancer xenotransplantation has confirmed that a lack of SIRT4 can accelerate the progression of cancer cell death in various cancers by regulating glutamine metabolism in the mitochondria or the mammalian target of rapamycin (mTOR) pathways (Csibi et al., 2013; Jeong et al., 2013). However, its role in breast cancer and OC seems to stand at the opposite end of the spectrum (Sun et al., 2019b; Wang et al., 2020). Du et al. (2020) have highlighted that SIRT4 negatively regulates SIRT1 expression by suppressing glutamine metabolism in mammary tumorigenesis, which implies the presence of competition functions within the sirtuin family.

Unlike the findings of Zhang J. et al. (2015), in our study, SIRT6 was not remarkably underexpressed in OC compared to its expression in normal ovary tissues, as observed *via* either RNA-seq or IHC staining intensity. According to our staining cohort, the IHC staining pattern of SIRT6 was more precise and suitable to be applied in clinical practice than that of SIRT4. In addition, SIRT6 DNA promoter methylation may explain why there is a trend of SIRT6 having more mRNA decreased patients in TCGA cohort, where SIRT4 does not. There is also a controversial report on the role of SIRT6 as a prognostic factor for OC. Bae et al. (2018) have demonstrated that SIRT6 can accelerate OC invasion capability by promoting beta-catenin translocation and shortening the survival time of OC patients. Poor prognosis was also identified in the study of Desantis et al. (2017). Meanwhile, more studies, including ours, have shown that SIRT6 acts as a tumor suppressor in human cancers (Van Meter et al., 2014; Desantis et al., 2017; Ioris et al., 2017). These inconsistent results may be explained by the genetic background of the research cohort and the statistical significance of the sample size, i.e., number of patients. Studies have revealed that SIRT4-mediated molecular mechanisms in OC are mainly focused on the mTOR, AMPK, and MAPK pathways (Betsinger and Cristea, 2019), whereas SIRT6 is widely associated with Notch-3/HIF and GLUT1 is involved in the Warburg effect. According to our findings, SIRT4 and SIRT6 are both related to the immune response of either the GO terms or KEGG immune-related diseases. However, their expressions were not significantly associated with T or B lymph cell infiltration. The indirect regulation of immune cells or the immune response environment should be investigated. More importantly, SIRT4 may negatively regulate intrinsic apoptotic signaling pathways *via* P53 class mediators, whereas SIRT6 may have the opposite functions. We also found that SIRT4 expression tends to increase with that of SIRT6, although the correlation is weak, indicating certain kinase activity alternation balance. The ratio of SIRT4 to SIRT6 is also a good biomarker for OS and PFS prediction. Applying the expression ratio (SIRT4/SIRT6) in both the KM Plotter database and the ICGC validation cohort, a higher ratio of SIRT4/SIRT6 suggests in both a worse clinical outcome. PPI network analysis also supports our hypothesis. For clinicians, the IHC staining pattern could be distinguished by pathologists for evaluating the expression levels of SIRT4 and SIRT6. In addition, there are not many reports on the application of the sirtuin family members as effective drug response biomarkers. To the best of our knowledge, our study is the first to discover that SIRT4 and SIRT6 can be considered as potential biomarkers for Plantin and Avastin treatment effectiveness for OC in addition to their role in the prediction of PFS for OC. Also, the limitation is that the AUC is not more than 0.7, which indicates that their value for anticancer drug response is still not satisfactory, as well as the ratio of SIRT4/SIRT6.

It is worth noting that there are many controversial reports on the dual role of the sirtuin family members or their functions in the same human disease model. It is more likely that the regulation network of these genes under each cancer model is still impervious. In addition, the sirtuin family members share the same protein acetylation sites as the NAD-dependent protein lysine-modifying enzymes. Potential competing functions also exist, which may affect the cancer model at an endogenous level, drawing conflicting conclusions. Although their biological functions are still under debate, there has been active research on designing chemical drugs for these enzymes, which may help to further clarify the role of a single gene in this family by blocking the activities of the other members in human cancer models (Parenti et al., 2014; Sociali et al., 2015).

Interpretation from the genomic network may help us understand the complex biological functions of the sirtuin family members at the endogenous level. Further confirmatory experiments should be carried out in order to validate our hypothesis. At the same time, we hope that our findings regarding SIRT4 and SIRT6 may provide new prospects for future drug research and clinical applications for OC patients. However, additional studies are essential to validate the concept for translational medicine.

## Data Availability Statement

The original contributions presented in the study are included in the article/Supplementary Material, further inquiries can be directed to the corresponding author/s.

## Ethics Statement

The studies involving human participants were reviewed and approved by the Harbin Medical University. The patients/participants provided their written informed consent to participate in this study.

## Author Contributions

HW and JL drafted the manuscript and analyzed the data. JL and RH performed figure preparation and data analysis. LF performed critical revision of the whole work. SY performed research design, manuscript drafting, and revision. All authors contributed to the article and approved the submitted version.

## Conflict of Interest

The authors declare that the research was conducted in the absence of any commercial or financial relationships that could be construed as a potential conflict of interest.

## Footnotes

1 http://www.cbioportal.org

2 http://kmplot.com/analysis/ovarian

3 https://www.proteinatlas.org/

4 http://www.linkedomics.org/login.php

5 https://david.ncifcrf.gov/

6 https://string-db.org/

## Supplementary Material

Supplementary Figure 1 **(A)** Kaplan–Meier survival plot of SIRT4 in OS (ICGC database). **(B)** Kaplan–Meier survival plot of SIRT6 in OS (ICGC database). **(C)** Kaplan–Meier survival plot using the ratio of SIRT4 and SIRT6 in OS. Red indicates higher expression than median; Green indicates lower expression than median (total *n* = 82).

Supplementary Figure 2 **(A)** Protein-protein interaction (PPI) network analysis for SIRT4 and SIRT6 with MCL clusters. Red nodes are for SIRT6, and green nodes are for SIRT4. **(B,C)** The OV cell line methylation association with mRNA RNA-seq expression for SIRT4 and SIRT6. Each dot represents an ovarian cancer cell line. Y axis indicates the correlation of promoter methylation.
