# Supplementary figures and images for: SIRT4 and SIRT6 Serve as Novel Prognostic Biomarkers With Competitive Functions in Serous Ovarian Cancer
Source: Front Genet. 2021 Jul 15;12:666630. doi: 10.3389/fgene.2021.666630 (PMC8320514; doi:10.3389/fgene.2021.666630)

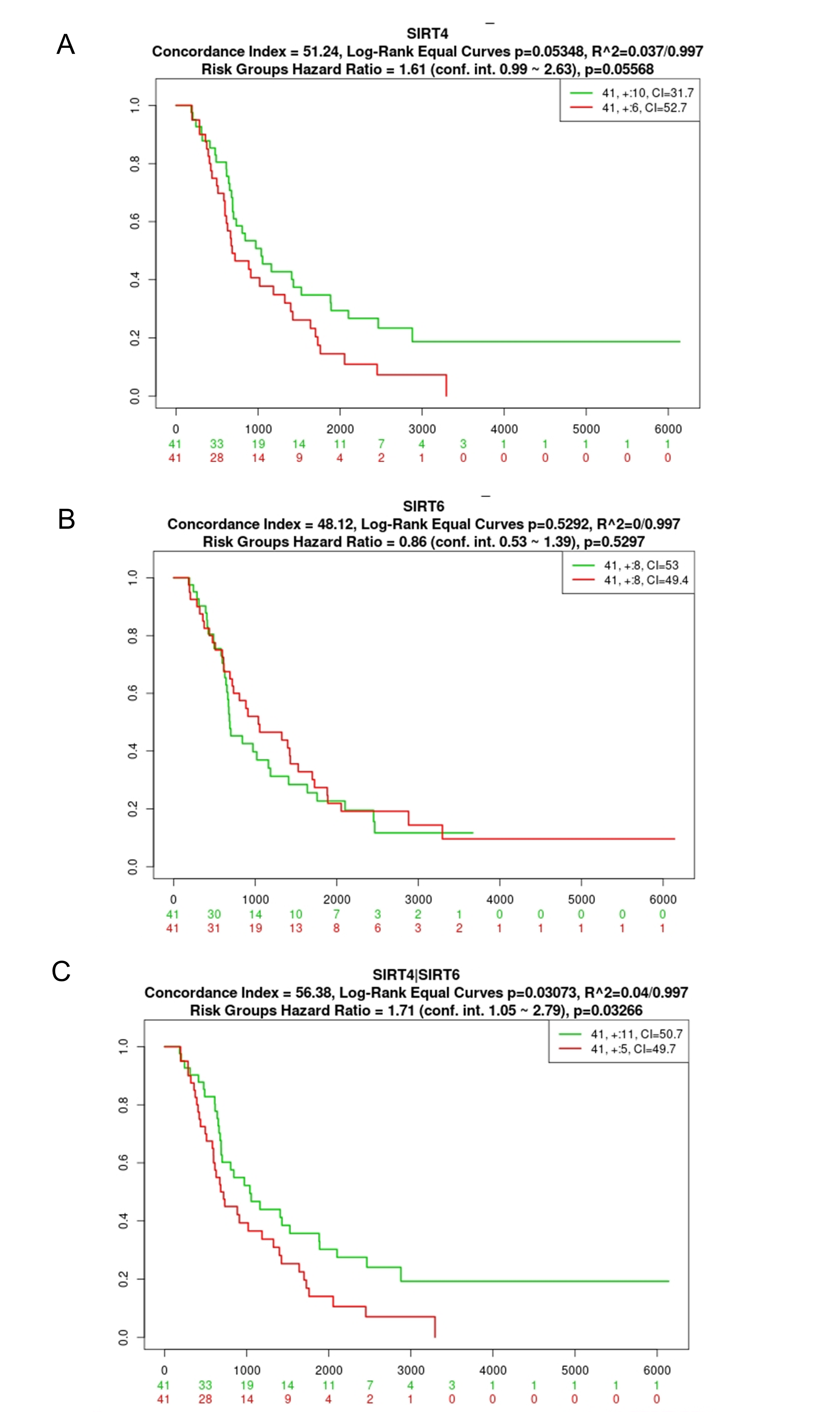

Supplement: Supplementary Figure 1 — (A) Kaplan–Meier survival plot of SIRT4 in OS (ICGC database). (B) Kaplan–Meier survival plot of SIRT6 in OS (ICGC database). (C) Kaplan–Meier survival plot using the ratio of SIRT4 and SIRT6 in OS. Red indicates higher expression than median; Green indicates lower expression than median (total n = 82). [file Image_1.TIF]

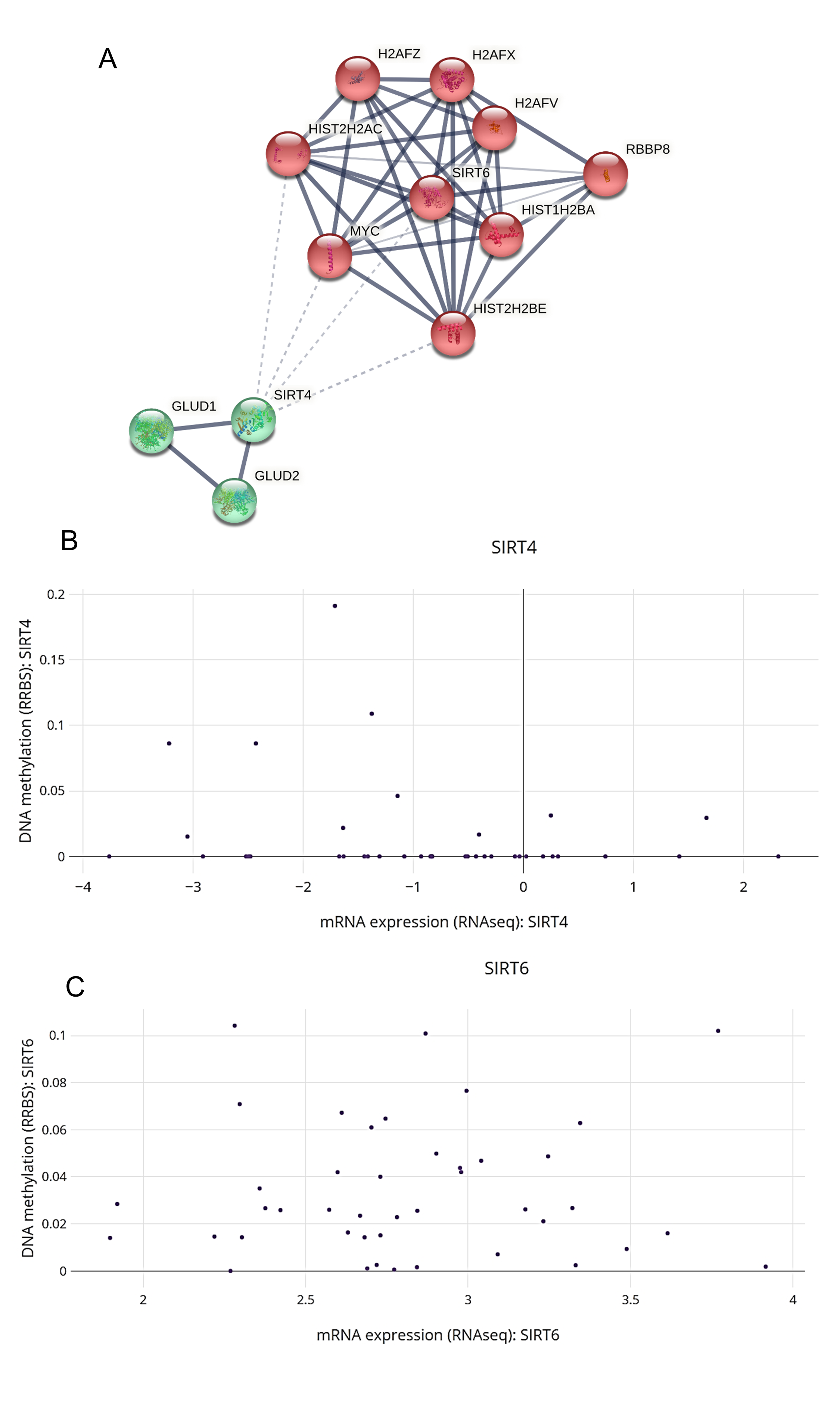

Supplement: Supplementary Figure 2 — (A) Protein-protein interaction (PPI) network analysis for SIRT4 and SIRT6 with MCL clusters. Red nodes are for SIRT6, and green nodes are for SIRT4. (B,C) The OV cell line methylation association with mRNA RNA-seq expression for SIRT4 and SIRT6. Each dot represents an ovarian cancer cell line. Y axis indicates the correlation of promoter methylation. [file Image_2.TIF]
